# CRISPR/Cas9-mediated knock out of ITGB6 in human OSCC cells reduced migration and proliferation ability

**DOI:** 10.1186/s13005-024-00437-x

**Published:** 2024-06-18

**Authors:** Maximilian Geyer, Fabian Geyer, Ute Reuning, Sarah Klapproth, Klaus-Dietrich Wolff, Markus Nieberler

**Affiliations:** 1 Department of Oral and Maxillofacial Surgery, Klinikum rechts der Isar der Technischen Universität München, D-81675 Munich, Germany; 2 Clinical Research Unit, Department of Obstetrics and Gynecology, Technische Universität München, D-81675 Munich, Germany; 3 Institute of Experimental Hematology, School of Medicine, Technische Universität München, D-81675 Munich, Germany

**Keywords:** Oral squamous cell carcinoma, Integrin beta 6, CRISPR/Cas9, Knock out

## Abstract

**Background:**

The treatment of oral squamous cell carcinoma (OSCC) remains challenging and survival rates have not been improved significantly over the past decades. Integrins have been recognized driving the cancer progression and high expression levels cause poor outcomes in patients afflicted with OSCC. Integrin αvβ6 and its subunit integrin beta 6 (ITGB6) were discovered to enhance the invasiveness by providing beneficial effects on downstream pathways promoting the cancer progression. The objective of this study was to establish a CRISPR/Cas9-mediated knock out of ITGB6 in the human OSCC cell line HN and investigate the effects on the migration and proliferation ability.

**Methods:**

ITGB6 knock out was performed using the CRISPR/Cas9-system, RNPs, and lipofection. Monoclonal cell clones were achieved by limiting dilution and knock out verification was carried out by sanger sequencing and FACS on protein level. The effects of the knock out on the proliferation and migration ability were evaluated by using MTT and scratch assays. In addition, in silico TCGA analysis was utilized regarding the effects of ITGB6 on overall survival and perineural invasion.

**Results:**

In silico analysis revealed a significant impact of ITGB6 mRNA expression levels on the overall survival of patients afflicted with OSCC. Additionally, a significantly higher rate of perineural invasion was discovered. CRISPR/Cas9-mediated knock out of ITGB6 was performed in the OSCC cell line HN, resulting in the generation of a monoclonal knock out clone. The knock out clone exhibited a significantly reduced migration and proliferation ability when compared to the wildtype.

**Conclusions:**

ITGB6 is a relevant factor in the progression of OSCC and can be used for the development of novel treatment strategies. The present study is the first to establish a monoclonal CRISPR/Cas9-mediated ITGB6 knockout cell clone derived from an OSCC cell line. It suggests that ITGB6 has a significant impact on the proliferative and migratory capacity in vitro.

## Introduction

Cancer of the oral cavity, predominantly oral squamous cell carcinoma (OSCC), is the 18th most prevalent type of cancer globally. With about 388,000 newly diagnosed cases and 178,000 related deaths estimated in 2020, it is an important public health issue, particularly in South Central Asia and developing countries. It is the leading cause of cancer-related mortality in India among men [1, 2]. Despite the advances in treatment strategies, the five-year survival rate of patients diagnosed with OSCC could not be improved lately [3–5]. Consequently, the identification of crucial key factors that facilitate invasion, migration, and metastasis of tumor cells is of significant importance for the development of novel diagnostic and treatment concepts [6]. Several surface proteins were identified, which appear to play a role in tumor progression, particularly with regard to proliferation and metastasis.

Integrins are a superfamily of heterodimeric transmembrane receptor proteins, composed of one α- and one β-subunit, which form 24 different integrin heterodimers [7, 8]. They mediate cell adhesion to proteins of the extracellular matrix (ECM) and are, moreover, capable of triggering bi-directional signal transduction across cell membranes, thereby contributing to the regulation of cell proliferation, invasion, migration, survival, and metastasis [9]. Integrin αvβ6 is a receptor recognizing an RGD motif (Arg-Gly-Asp) in distinct proteins, including fibronectin, vitronectin, tenascin-C, and the latency associated peptide (LAP) of transforming growth factor-β1 (TGFβ1) [10–13]. The expression of αvβ6 in normal, healthy tissue is low to absent, whereas increased levels are detectable in wound healing, developing tissue, fibrosis, and distinct cancer entities, such as OSCC [12, 14–18]. Thereby, the presence of αvβ6 is related to a poorer patient prognosis in various cancer types, including OSCC [19]. ITGB6 is present just in the given heterodimer, connected to αv, whereas subunit αv can also be associated with other β-subunits. Hence, the β6-subunit (ITGB6) acts as the rate-limiting factor for the formation of the heterodimer αvβ6, whose expression is regulated by different transcription factors [18]. Consequently, by targeting ITGB6, αvβ6 is the only heterodimer that is affected [20].

Integrin αvβ6 is implicated in a multitude of different (tumor) cell biologically relevant functions. These include cell adhesion, migration, invasion, and proliferation [13]. Several studies have demonstrated its beneficial effect to the migration, invasion, and proliferation potential in OSCC by upregulation of the two matrix metalloproteinases MMP-3 and MMP-9, or protecting cells of anoikis [13, 21–24]. Increased expression of αvβ6 is particularly observed at the invasive tumor front, where it contributes to the invasive potential of tumor cells [6, 13]. Therefore, integrin αvβ6 interacts with pro-invasive membrane proteins at the tumor edge, e.g. the TGFβ type II receptor and the urokinase receptor, thereby promoting the proteolytic actions of TGFβ1 at the invasive tumor front [20, 25].

Hence, ITGB6 represents a potential target, which may be exploited for clinical tumor imaging, therapy, theranostics, and as a prognostic marker, potentially resulting in an earlier diagnosis and intervention [19, 26–29].

The aim of the present study was to determine the impact of ITGB6 expression on the overall patient survival and outcome, as well as the proliferative and migratory capacity of OSCC cells by depleting cells of ITGB6 expression. For this purpose, we established a stable knock out (KO) cell subclone of the subunit ITGB6 involving the CRISPR/Cas9 system, derived from the OSCC cell line HN. After the verifying the ITGB6-KO by flow cytofluorometry, the generated cell clones were compared to αvβ6-harboring wild type (WT) cells with respect to their growth and motile activity.

## Materials and methods

### In silico TCGA data analysis

The web-based cancer genomics resource database CBioPortal (https://www.cbioportal.org, accessed on 25th August 2023) was used to gather data regarding the mRNA expression levels of ITGB6 in patients afflicted with OSCC [30, 31]. The Head and Neck Squamous Cell Carcinoma TCGA Firehouse Legacy data set, comprising 530 tumor samples, was utilized. By limiting the analysis to samples with the designation “Primary tumor” for sample type, available T-stage, and the primary tumor sites “Oral Tongue”, “Oral Cavity”, “Floor of mouth”, “Base of tongue”, “Buccal Mucosa”, “Alveolar Ridge”, “Hard Palate”, and “Lip” in order to select OSCC for our focus, the number of samples an patients was reduced to 335. The expression levels of mRNA were normalized to z-scores relative to diploid samples (RNA Seq V2 RSEM). Two groups were built (1st and 4th quartile) based on the ITGB6 mRNA expression levels (low expression z-score: -1.04 - -0.64; high expression z-score: 0.46–6.39). The total number of patients included in the study patients decreased to 165 (82 in group “low expression”, 83 in group “high expression”).

### Cell culture

The used OSCC cell line “HN” was gained from a lymph node metastasis of a moderately differentiated squamous cell carcinoma of the soft palate (DMSZ; ACC 417). The derivation of the cell line from a metastatic cancer is of great importance in evaluating the impact of ITGB6 on the metastatic potential. The cells were maintained in Dulbecco´s Modified Eagle´s Medium (DMEM) (Sigma Aldrich, St. Louis, Missouri, USA) supplemented with 10% (v/v) fetal calf serum (FCS) (Sigma Aldrich, St. Louis, Missouri, USA) and cultured in accordance with previously described methodology [32].

### Genome editing using CRISPR/Cas9

For detailed information on the protocol, exact amounts of applied components and the entire workflow, please refer to Geyer et al. [32].

Gene editing was performed using RNPs (ribonucleoprotein complexes) containing a gRNA and Cas9 protein, transfected by lipofection. In this study the guide sequence “gRNA1” (GCTAATATTGACACACCCGA) on genomic position Chr2. 160,174,011, targeting the human ITGB6 locus on chromosome 2 with a positioning of the Cas9 cut site on the fifth exon, was used. The sgRNA was assembled by heating up equimolar amounts of the guide sequence (Alt-R CRISPR/Cas9 crRNA; IDT, Coralville, Iowa, USA) and Alt-R CRISPR/Cas9 tracrRNA (IDT, Coralville, Iowa, USA) for 5 min at 95 °C. Overall, for each well (24-well format) 480 ng gRNA and 250 ng Cas9 protein were used for lipofection.

24 h prior to transfection, 50,000 cells were seeded per well on a 24-well-plate, in order to achieve a confluency of 50–60% at the time of transfection. Transfection was conducted in a 24-well format using Lipofectamine CRISPRMAX (Invitrogen, Waltham, USA) for a period of 48 h. For the complete RNP assembly, TrueCut Cas9 v2 (Thermofisher, Waltham, MA, USA) was employed. DNA isolation was performed directly from cells, using the Phire Tissue PCR Master Mix (Thermofisher) in the “Dilution and storage protocol” according to the manufacturers’ protocol. Thereof 1 µl was used for amplification of genomic DNA by PCR prior to sequencing. Two flanking primers were designed (fwd: 5`-GTTGCAGAGTCAGGCCCTTTAG-3`; rev: 5`-GGACAGTCCCCATTTCAACATG-3`) to obtain amplicons of about 500 bp (250 bp up- and downstream of the Cas9 cut site) for *Sanger* sequencing. PCR was performed according to the “Phire Tissue PCR Master Mix” (Thermofisher) manufacturers protocol. For details on the cycling conditions, please refer to Geyer et al. [32].

DNA purification was conducted using the qiaQuick DNA purification Kit (Qiagen, Hilden, Germany), in accordance with the suggested Kit protocol. The purified PCR products were then subjected to sequencing at Eurofins Genomics (Ebersberg, Germany) at a minimum concentration of 5 ng/µl. The forward PCR primer was used as the sequencing primer. Sequence analysis was done with ICE (Synthego, Redwood City, USA; https://ice.synthego.com) [33].

### Isolation of a monoclonal knockout cell clone

For the isolation of ITGB6 KO cell clones derived from a single cell, limiting dilution was used. For this, cells were counted and diluted in cell culture medium to a concentration of 5 cells / 1 ml. Subsequently, 100 µl of the solution were added to each well of a 96-well plate, resulting in a concentration of 0.5 cells per well. The plates were incubated at 37 °C and 5% (v/v) CO_2_ for 4 to 7 days. At this point, the first colonies could be identified under the microscope (100 x magnification). Wells with more than one colony were discarded. The remaining wells were then further cultured under the given conditions. For analysis of the clones, please refer to the aforementioned steps.

### Analysis of protein expression using flow cytofluorometry

Cells were stained for ITGB6 by using standard procedures for flow cytometry.

For this, cells were incubated for 10 min with a Fc receptor-blocking antibody (BD Biosciences, Heidelberg, Germany) and iFlour 840 maleimide live-dead stain (AAT Bioquest, Pleasanton, CA,

USA*).* Subsequently the cells were stained with an APC-labeled anti-ITGB6 antibody (Miltenyi Biotec, Bergisch Gladbach, Germany; Catalog#: 130-111-454) for 30 min in phosphate-buffered saline (PBS) supplemented with 2% (v/v) FCS and 2 mM EDTA. Analysis was performed using a Cytoflex LX flow cytometer (Beckman Coulter, Brea, CA, USA).

### MTT proliferation assay

The extent of cell proliferation was quantified using the (3-(4,5-dimethylthiazol-2-yl)-2,5-diphenyltetrazolium bromide (MTT) assay [34]. For this purpose, HN cells were plated at a density of 4,000 cells/100 µl/96-well and cultured for 24 h.

Prior to spectrophotometric measurement at day one to five, 20 µl of MTT (5 mg/ml) were added to each well and incubated for 2.5 h at 37 °C and 5% (v/v) CO_2_. Subsequently, the solution of culture medium and MTT was carefully aspirated, and 100 µl DSMO added to each well.

The 96-well cell culture plates were then gently agitated in order to completely dissolve all blue formazan crystals. By use of a Multiskan™ Fc photometer (Thermo Fisher Scientific, Waltham, USA), the absorbance of the solution was spectrophotometrically determined at 570 nm as measure of cell numbers. Measurements were done in triplicate and in three independent experiments.

### Wound scratch assay

The migratory activity of cells was analyzed by wound scratch assays.

For this, 100,000 cells/well were seeded in 800 µl FCS-free cell culture medium on 24-well cell culture plates in order to minimize the effects of proliferation over migration and incubated for 24 h. The culture medium was then aspirated and a wound scratch set by use of a 200 µL pipette tip. Thereafter, cells were washed thoroughly in PBS and fresh FCS-free cell culture medium was added. Microscopic images (Zeiss, Oberkochen, Germany) were taken directly after wounding the cell monolayers (start) as well as after every other 4 h until the wound gap closure was visible. The extent of wound gap closure over time was analyzed by ImageJ (https://imagej.nih.gov/ij) using the “Wound Healing Size Tool”, established by Suarez-Arnedo et al. [35].

### Statistical analysis

Each experiment was repeated at least three times and was carried out in triplicate. The data were expressed as mean ± SD. Overall survival was analyzed using the Kaplan-Meier method and survival difference was compared by log-rank test. Statistical analysis was conducted using GraphPad Prism (Version 9.5.1). The differences between two groups were determined by Student´s t test, and the differences among multiple groups were determined by one-way Anova. The clinical outcome parameters were compared using the Chi-Squared test. The time from diagnosis to death by any cause was defined as OAS. The results were assumed to be significant if *p* < 0.05.

## Results

### ITGB6 expression decreases overall survival in patients afflicted with OSCC

**Fig. 1:**
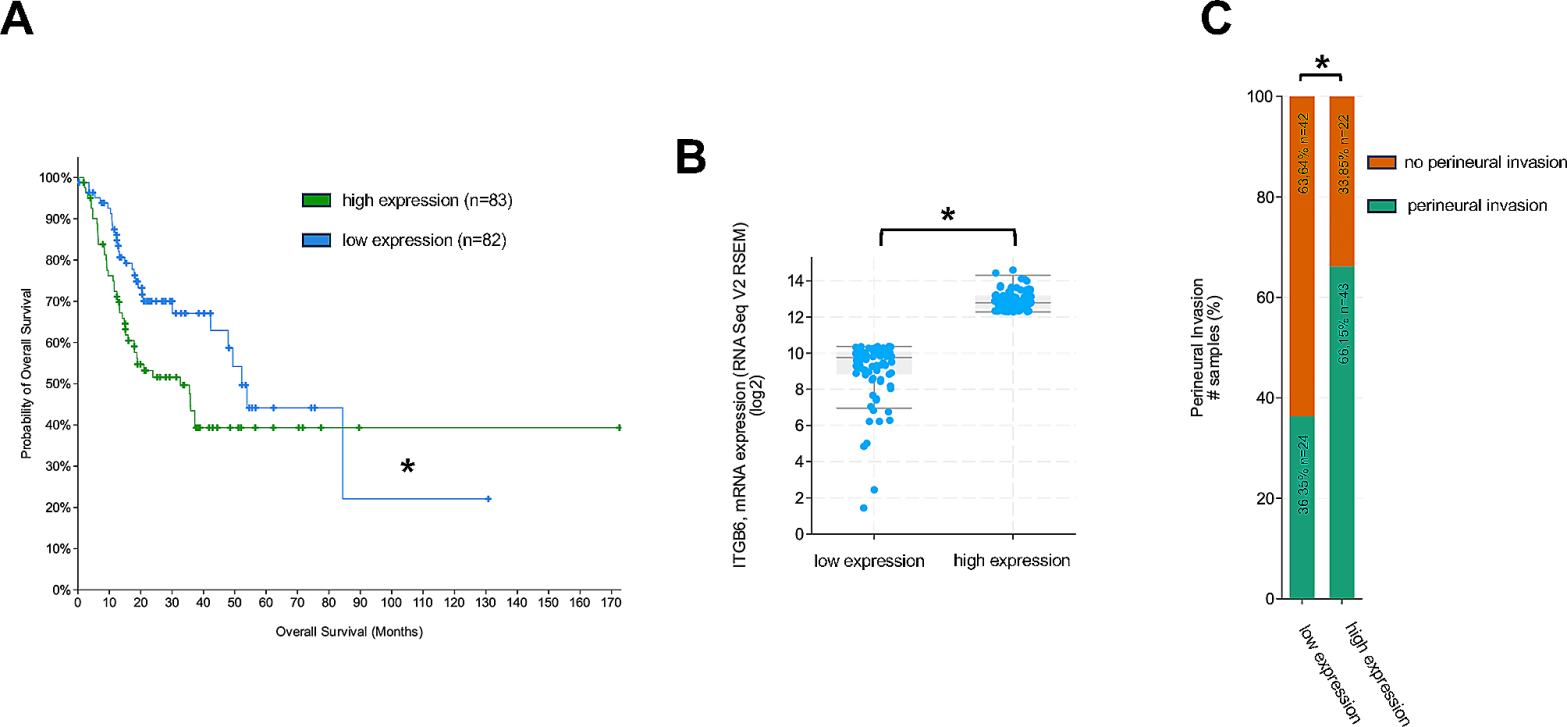
Impact of ITGB6 mRNA expression on patient outcome. **A** Kaplan-Meier plot on the overall survival of patients afflicted with OSCC. Comparison of “high expression” (*n* = 83) mRNA ITGB6 levels and “low expression” (*n* = 82) mRNA ITGB6 levels. Significant longer overall survival of the “low expression” group, * *p* = 0.0355. **B** Plot of the mRNA expression levels of the group “low expression” and “high expression”. Significant difference in the mRNA expression levels of ITGB6, * *p* < 0.000001. **C** Comparison of perineural invasion in both groups. Significant increased perineural invasion in the “high expression” group, * *p* = 0.001214

Initially, we investigated the impact of ITGB6 expression on the survival of patients diagnosed with OSCC. In silico analysis of data from the specified groups by CBioPortal (https://www.cbioportal.org) was used. Kaplan-Meier analysis revealed a significant impact of ITGB6 mRNA expression levels on the overall patient survival. The median OAS for the group “low ITGB6 expression” was 52.27 months (*n* = 82), whereas the group “high ITGB6 expression” showed an OAS of 32.62 months (*n* = 83, *p* = 0.0355) (Fig. 1A). The ITGB6 mRNA expression levels of both groups are shown in Fig. 1B. Taken together, a high ITGB6 expression was found to be correlated with a reduced survival time of OSCC patients.

As perineural invasion (PNI) is correlated with treatment failure and decreased overall survival of OSCC patients, we analyzed the impact of ITGB6 expression on PNI in both groups [36].

In addition to the reduction in survival outcome, further analysis revealed a significantly higher rate of PNI of the “high ITGB6 expression” group versus the “low ITGB6 expression” group (Fig. 1C, *p* = 0.001214), whereas no significant differences in positive N-status were discovered.

### CRISPR/Cas9 knockout of ITGB6 in HN cells

The influence of ITGB6 within these findings was then addressed in vitro, by establishing an ITGB6 KO in OSCC cells using the CRISPR/Cas9 system.

According to Geyer et al., the overall protocol and efficiency were optimized [32]. Due to the high editing efficiencies (74 ± 7% INDEL%; 68 ± 4% KO score), the approach with one gRNA was used and limiting dilution was possible to generate a monoclonal cell line. Initially, the obtained clones were analyzed by Sanger sequencing approximately 21 days after single cell seeding and have been expanded. The given primers flanking the gRNA binding site were used to amplify the region around the Cas9 cut site on the ITGB6 locus. Those clones that suggested a complete knockout of ITGB6 were then further assessed on the protein level by FACS.

In total, 12 clones were picked. Four of these clones were found to be wild type following sequence analysis. Four other obtained cell clones indicated a KO of just one allele, while the second allele showed the wild type. In total, four clones were identified as potential KOs of ITGB6 by analysis with the ICE online tool (Synthego, Redwood City, USA; https://ice.synthego.com). The sequence traces of the first analyzed cell clone “ITGB6KO #1” and the corresponding WT cells are shown in Fig. 1A, including the position of the Cas9 cut site. Following the consideration of the given results, we proceeded to confirm the KO first on cell clone “ITGB6KO #1” using cytofluorometry.

**Fig. 2:**
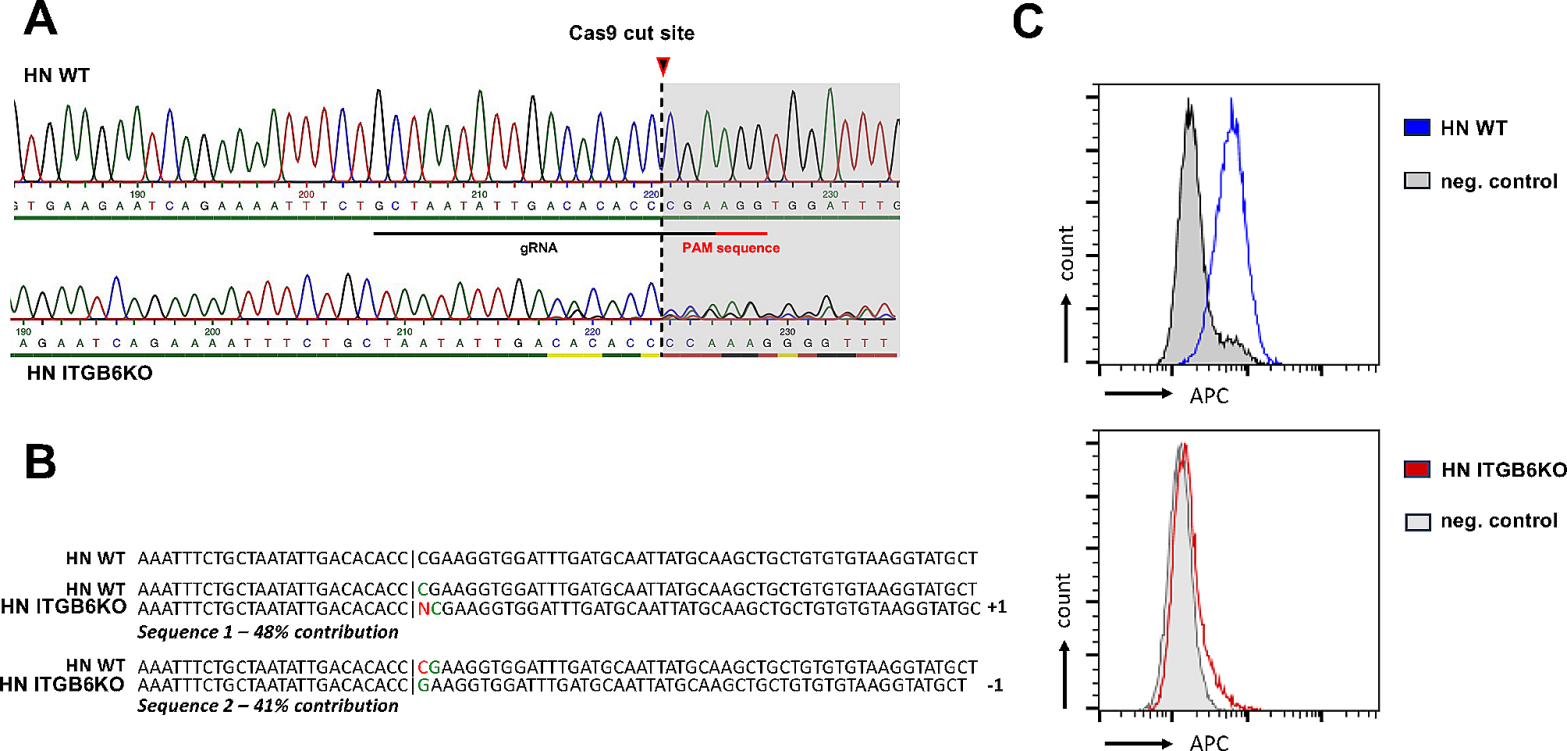
CRISPR/Cas9-mediated knockout of ITGB6 in HN cells. **A** Sanger sequencing of HN WT (top) and HN ITGB6KO (bottom). Traces and sequences are presented. PAM sequence, gRNA binding and Cas9 cut site is marked. **B** Sequencing data of HN ITGB6KO analyzed by ICE online tool (Synthego). Decomposition of the found sequences with the individual contributions are presented (Sequence 1 1: 48%; Sequence 2: 41%). Sequences with contributions ≤ 2% are considered as background signal and are not shown. Sequence 1 shows an insertion of one base (marked in red color) (+ 1). Sequence 2 indicates a deletion of 1 base (-1) (deleted base shown in red color in the WT sequence). Overall, a strong indication of a heterozygous KO of ITGB6 with frameshift mutations on both alleles is presented. **C** Verification of ITGB6 gene KO of HN ITGB6KO on the protein level by FACS analysis. HN WT cells present with a homogenous ITGB6 expression, whereas HN ITGB6KO indicates a loss of ITGB6 expression

In ICE analysis, ITGB6KO #1 strongly indicated a deletion of one base on one allele and an insertion of one base on the other, resulting in a frameshift mutation on both alleles, consequently suggesting a complete KO in this clone. Manual alignment of the sequences underlined these results (Fig. 2B). The sequencing results for ITGB6KO #1 were verified on the protein level by flow cytofluorometry.

As a control, HN WT cells were used, with a homogenous expression of ITGB6, whereas no positive cells with ITGB6 expression were found for HN-ITGB6KO #1, from here on now referenced as HN ITGB6KO (see in Fig. 2C). The distinct cell count showed 5,871 positive cells for the WT cells (neg. control 1769) and 1,471 for HN ITGB6KO (neg. control 1,239).

### Characterization of the impact of an ITGB6 KO in HN cells on their proliferative and migratory capacity

Due to its functional implications in migration and proliferation of cancerous cells, ITGB6 represents an important target structure for the development of novel strategies for the diagnosis and treatment of epithelial neoplasms [18]. In order to further address these tumor biologically relevant abilities as a function of αvβ6 expression, we assessed the behavior of the HN ITGB6KO cells in comparison to WT cells in functional in vitro assays.

**Fig. 3:**
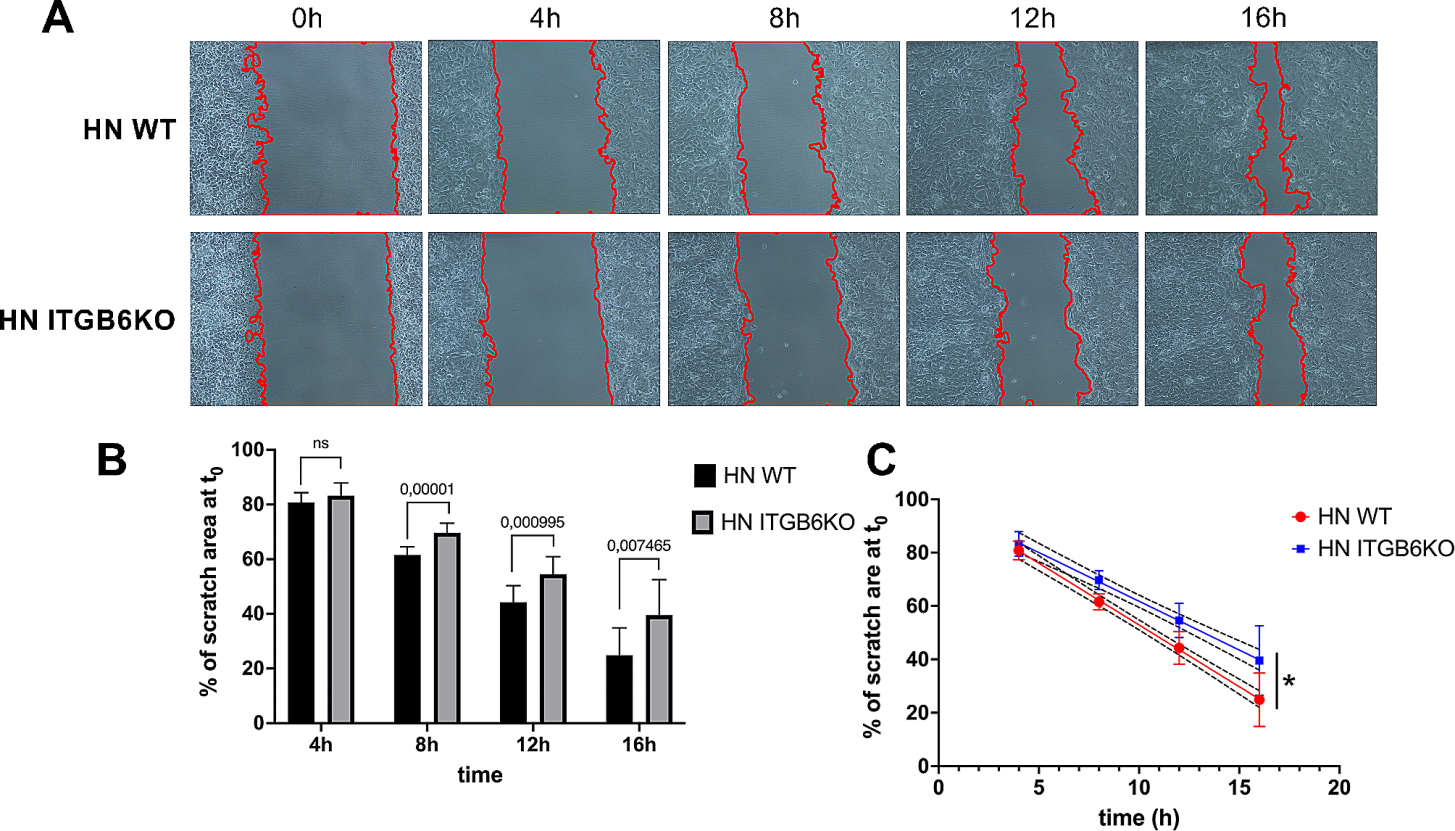
Migration of HN WT and HN ITGB6KO. **A** The migratory capacity of HN WT and HN ITGB6KO cells was compared by wound scratch assays. Microscopic images of wound gap closure were taken at every 4 h after setting the wound scratch (100 x magnification). Wound gaps marked with red line. **B** Quantification of the closure of the wound scratch by calculating the percentage cell free surface area in relation to the initial scratch at start. HN WT cells migrated significantly faster than HN ITGB6KO. P values are shown above the bars. **C** Linear regression of the closure of the wound scratch area. 95% CI marked with dotted lines. Both slopes differ significantly. * *p* = 0.0042

To determine the migratory behavior of HN ITGB6KO cells in comparison to HN WT cells, wound scratch assays were performed. The extent of wound gap closure was calculated relative to the wound gap area at the start of the experiment and followed over every four hours. In general, WT cells migrated faster than HN ITGB6KO cells (Fig. 3A). The first significant differences were observed already within eight hours after setting the wound scratch (*p* = 0.00001). After 16 h, total wound gap closure was noticeable for both cell variants. A linear regression analysis of the data collected (Fig. 3C) disclosed a significant difference of the slopes between WT and HN ITGB6KO cells (HN WT R^2^ 0.922; HN ITGB6KO R^2^ 0.828; *p* = 0.0042).

In conclusion, the ITGB6 KO in HN resulted in a significantly reduced migratory capability when compared to WT cells.

**Fig. 4:**
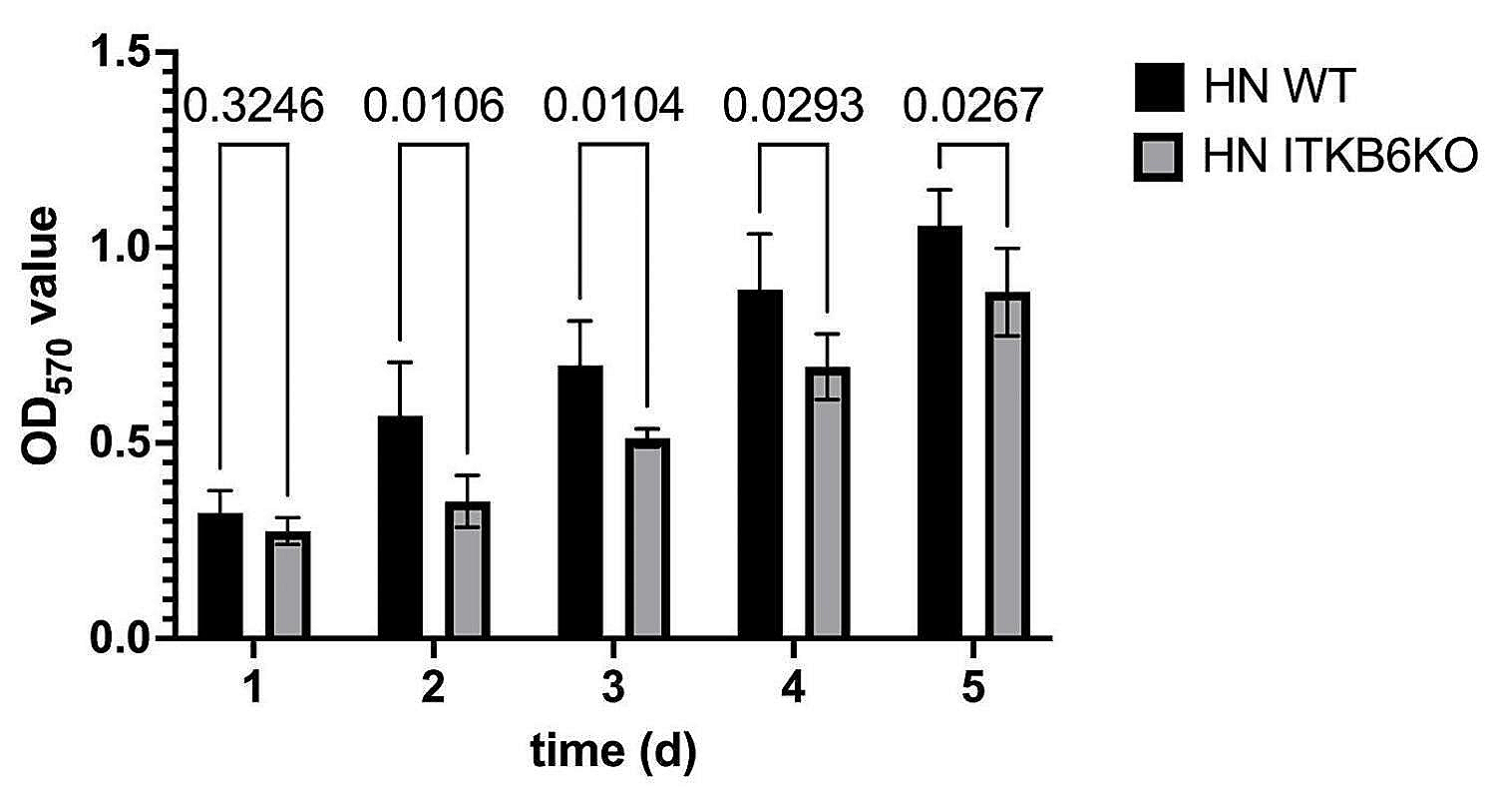
Determination of HN cell proliferative activity as a function of αvβ6 expression. The cell proliferative activity was measured by MTT assays over 5 days. The relative absorbance (OD_570_ value) is presented as measure of cell numbers. HN WT cells proliferated significantly faster than HN INTGB6KO cells. P values are shown above the bars

Another hallmark of cancer is given by tumor growth. In order to analyze the contribution of ITGB6 to the proliferative activity of HN cells, we compared the growth of HN WT cells to HN ITGB6KO cells. The proliferative ability of HN WT cells was found to be significantly higher than that of HN ITGB6KO cells, with a significant difference already evident at day 2 of the MTT-measurements (Fig. 4A). The calculation of the doubling times showed 42 h 45 min for WT cells and 53 h 21 min for HN ITGB6KO cells.

These results collectively indicated that ITGB6 significantly contributes to HN cell proliferative activity.

## Discussion

ITGB6, respectively αvβ6, is well known for being highly expressed in various cancer types of epithelial origin, such as carcinomas of the lung, breast, stomach, liver, skin, cervix, salivary glands, and oral mucosa [19, 37, 38]. Almost 100% of OSCC tissues are αvβ6-positive as proven in various studies. In contrast, ITGB6 is absent in normal buccal mucosa [37, 39]. Increased expression of αvβ6 is associated with a higher invasiveness of OSCC [40]. It is linked to a poor patient prognosis and survival due to its involvement in tumor progression and metastasis, whereby it promotes tumor-relevant biological functions, such as cell proliferation and migration / invasion [18, 19, 41].

In the present in vitro study, we asked whether ITGB6 has a significant impact on the migratory potential and proliferative activity of OSCC cells, consequently, contributing to overall patient survival and clinical parameters associated with an altered migratory and proliferative ability, such as overall survival and perineural invasion. To this end, we conducted an in silico analysis to explore the mRNA levels of ITGB6 in patients afflicted with OSCC. Our findings demonstrated a correlation between high ITGB6 expression and reduced survival of OSCC patients and perineural invasion. This highlights the clinical significance of this integrin subtype for patient outcome, proposing it as an attractive, cell surface available, and potent target structure for novel treatment strategies in OSCC.

A stable monoclonal ITGB6 KO cell clone was generated using the CRISPR/Cas9 genome editing system for studies on a molecular level. Sequence analysis of HN ITGB6KO indicated a deletion of one base on one allele and a one base insertion on the other, resulting in a frameshift mutation on both alleles. The loss of ITGB6 expression on the protein level was confirmed by flow cytofluorometry.

By use of this cell model system, cell biological in vitro assays were performed to investigate the migratory capability of HN cells as a function of ITGB6 expression by wound scratch assays. Regarding the impact of ITGB6 on the migratory potential of HN cells, we found a significantly reduced migratory capacity of HN ITGB6KO in comparison to WT cells, suggesting that ITGB6, respectively αvβ6, is a driver of cell migration. These findings are in good accordance with other studies using tumor cells of various cancer entities. In fact, it has been previously reported, that αvβ6 is capable of promoting cancer cell migration upon binding to and adhering to protein ligands of the ECM, such as fibronectin and vitronectin [41]. Furthermore, by intracellular signaling involving the TGF-β1 and Raf-ERK-MAPK pathways, it is implicated in the activation of matrix metalloproteases, such as MMP2, MMP3, and MMP9, which facilitates ECM remodeling required for cell invasion of the ECM [42–44]. Similarly, Soejima et al. also showed by trans-well and wound scratch assays a significantly lower migratory capacity of cholangiocarcinoma cells when ITGB6 was lacking. The invasive potential was also decreased [45]. Upon silencing ITGB6 in cholangiocarcinoma cells through siRNA, Zequn et al. confirmed the contribution of αvβ6 to the motile cell activity. Conversely, they observed an induced migratory activity following ITGB6 overexpression [46]. Zhao et al. discovered that the migration of AGS gastric carcinoma cells was inhibited following a αvβ6 knock down by siRNA and blockade of β6 by function blocking antibodies [47]. This was also observed for cervical cancer cells upon silencing of ITGB6 through inactivation of the JAK/STAT3 signaling pathway [48].

Besides the migratory ability of cancer cells, their increased proliferative activity also contributes to tumor progression and poor survival rates of OSCC patients. By ITGB6 KO, we observed an early and significantly reduced proliferative capacity of HN cells, which was accompanied by a decreased cell viability when compared to WT cells. This is in line with several other in vitro studies [23, 49, 50]. An association between increased αvβ6 expression and a higher proliferation rate of tumor cells of other cancer entities had been documented [18, 19]. As such, a reduced proliferation and migration rate of lung cancer cells (A549 and H460) was shown by Yan et al. following αvβ6 silencing by siRNA [51].

Overall, our findings indicate that the expression level of ITGB6 is associated with tumor progression and invasiveness in OSCC (e.g., perineural invasion). Thus, ITGB6 represents not only a valuable prognostic factor in OSCC, but also an attractive target for therapy and diagnosis (theragnostic) in different cancer entities, including OSCC. For the latter this is of special importance since new treatment options are urgently demanded, because so far, OSCC patients are mostly treated by surgical resection, radiotherapy, chemotherapy, or a combination of these regimens, depending on the tumor stage [52]. Unfortunately, the improvement of disease outcome and enhanced patient survival is far from being satisfying [3].

Currently, chemotherapy faces drawbacks from non-specific cell death and the resulting side effects in OSCC [52]. The advantage of ITGB6 as a target structure lies in its exclusive expression in certain wound healing conditions and cancer, associated with its absence in normal adult epithelia [37]. Given its expression (in particular at the invasive tumor front) in greater than 95% of the cases, it is an appropriate target for the imaging of cancerous cells for diagnostic and therapeutic purposes, particularly in OSCC [18, 37].

There are several approaches for the usage of ITGB6 and αvβ6 as target structures in cancer. Various studies have already designed and developed different peptidic small molecule αvβ6 antagonists, competing with ECM ligands for αvβ6 binding [18, 29, 53, 54] which are currently explored for their use for diagnostic imaging or intraoperative guidance. Moreover, other studies showed promising results by lowering metastasis by using antibodies against αvβ6 [37, 42]. There are several drugs in various clinical stages that target αvβ6 as potential treatment options. Consequently, there is a need for cell models for further research, most notably for OSCC.

This study represents the inaugural report on the establishment of an OSCC ITGB6 KO cell clone, which exhibited reduced migratory and proliferative activity compared to WT cells displaying αvβ6 on their cell membranes. Thus, HN ITGB6KO may serve as a valuable cell model for future investigations of the tumor-biological effects of ITGB6 in OSCC.

## Conclusions

In conclusion, this study reports the successful in vitro establishment of a monoclonal CRISPR/Cas9-mediated ITGB6 knockout cell clone derived from the OSCC cell line HN. Evaluation of the migratory and proliferative capacity revealed a significantly reduced potential when compared to the wild type. This underlines the importance of ITGB6 on OSCC progression and prognosis. In addition, the impact of ITGB6 mRNA expression levels on overall patient survival and perineural invasion are shown (in silico TCGA analysis). Furthermore, the obtained knock out cell clone can serve as a cell model for additional research in the future.

## Data Availability

No datasets were generated or analysed during the current study.
